# Interactions between Dorsal and Ventral Root Stimulation on the Generation of Locomotor-Like Activity in the Neonatal Mouse Spinal Cord

**DOI:** 10.1523/ENEURO.0101-16.2016

**Published:** 2016-07-08

**Authors:** Avinash Pujala, Dvir Blivis, Michael J. O’Donovan

**Affiliations:** 1 Janelia Farms Research Campus, Ashburn, Virginia 20147; 2 Section on Developmental Neurobiology, NINDS, NIH, Bethesda, Maryland 20892

**Keywords:** central pattern generator, dorsal root, locomotion, spinal cord, ventral root

## Abstract

We investigated whether dorsal (DR) and ventral root (VR) stimulus trains engage common postsynaptic components to activate the central pattern generator (CPG) for locomotion in the neonatal mouse spinal cord. VR stimulation did not activate the first order interneurons mediating the activation of the locomotor CPG by sacrocaudal afferent stimulation. Simultaneous stimulation of adjacent dorsal or ventral root pairs, subthreshold for evoking locomotor-like activity, did not summate to activate the CPG. This suggests that locomotor-like activity is triggered when a critical class of efferent or afferent axons is stimulated and does not depend on the number of stimulated axons or activated postsynaptic neurons. DR- and VR-evoked episodes exhibited differences in the coupling between VR pairs. In DR-evoked episodes, the coupling between the ipsilateral and contralateral flexor/extensor roots was similar and stronger than the bilateral extensor roots. In VR-evoked episodes, ipsilateral flexor/extensor coupling was stronger than both the contralateral flexor/extensor and the bilateral extensor coupling. For both types of stimulation, the coupling was greatest between the bilateral L1/L2 flexor-dominated roots. This indicates that the recruitment and/or the firing pattern of motoneurons differed in DR and VR-evoked episodes. However, the DR and VR trains do not appear to activate distinct CPGs because trains of DR and VR stimuli at frequencies too low to evoke locomotor-like activity did so when they were interleaved. These results indicate that the excitatory actions of VR stimulation converge onto the CPG through an unknown pathway that is not captured by current models of the locomotor CPG.

## Significance Statement

In 2005, we showed that stimulation of motor axons can activate the neural circuitry for locomotion in the neonatal mouse spinal cord. This was a surprising result because motoneurons are thought to be purely output elements of the spinal cord. Here we show that motor axons reach the locomotor circuitry by a different pathway to that used by sensory axons. Despite this difference, both types of stimulation activate common locomotor circuitry. An understanding of how motor axons activate these circuits will provide novel insights into the organization of locomotor networks.

## Introduction

In isolated preparations of the neonatal rodent spinal cord, locomotor-like activity can be initiated by bath-applied drugs, or by electrical stimulation of the dorsal roots (DRs; Whelan et al., 2000; Delvolvé et al., 2001) or the brainstem (Zaporozhets et al., 2004). Although the mechanisms responsible for such activation are not clear, some progress has been made in understanding how lumbar locomotor networks are activated by stimulation of sacrocaudal afferents (SCAs). Lev-Tov and his collaborators have shown that SCA activation of the lumbosacral locomotor central pattern generator (CPG) is mediated by a set of relay interneurons that project to the lumbar locomotor networks both directly and indirectly through the white-matter tracts (Strauss and Lev-Tov, 2003; Etlin et al., 2010). These relay interneurons are innervated by sacral sensory fibers, including VGluT1^+^ muscle spindle afferents and VGluT2^+^ afferents. During SCA stimulation, calcium imaging has demonstrated that these neurons deliver both a tonic and a rhythmic drive to the lumbar segments (Etlin et al., 2013). When their ascending axons are interrupted using white-matter lesions, SCA stimulation failed to activate the generator indicating their critical role in mediating the effects of dorsal root stimulation (Etlin et al., 2010, 2013).

Stimulation of ventral roots (VRs) can also trigger locomotor-like activity (Mentis et al., 2005), modulate the frequency of drug-induced locomotor rhythms (Machacek and Hochman, 2006) and entrain disinhibited bursting (Machacek and Hochman, 2006; Bonnot et al., 2009). These effects persist in the presence of cholinergic antagonists but are blocked or reduced by ionotropic or metabotropic glutamatergic antagonists (Mentis et al., 2005; Bonnot et al., 2009). Beyond this, nothing is known about how VR stimulation triggers locomotor-like activity. Given that both dorsal and ventral root excitation of the locomotor generator are mediated by glutamatergic mechanisms, we investigated the extent to which they share common mechanisms for activating the generator and if the two types of stimulation activate the same generator. We investigated this by pairing or interleaving stimulus trains to dorsal and ventral roots and establishing their ability to trigger locomotor-like activity. We also performed experiments to address the possibility that the effects of VR stimulation might be mediated by afferents within the VRs (Windle 1931; Coggeshall 1980).

## Materials and Methods

### Surgical procedures

Experiments were performed on Swiss Webster neonatal mice (Taconic Laboratory; P0–P4) of either sex. The mice were decapitated and eviscerated and the remaining tissue was placed in a dissecting chamber and continuously superfused with an artificial cerebrospinal fluid (ACSF; concentrations in mm: 128 NaCl, 4 KCl, 1.5 CaCl_2_, 1 MgSO_4_, 0.5 NaH_2_PO_4_, 21 NaHCO_3_, 30 d-glucose) bubbled with 95% O_2_-5% CO_2_. A ventral laminectomy exposed the cord, which was then transected between the 5th and 7th thoracic segments and removed from the vertebral column together with the attached roots and the cauda equina. In some experiments, the cord was not transected to preserve connections with the brainstem. During the dissection, the ACSF was initially at a temperature of 6–7°C and was progressively allowed to warm to 23–25°C (room temperature) before transfer of the cord to the recording chamber.

### Electrophysiology

#### Recording

Electrical activity from motoneurons was recorded with tight-fitting plastic suction electrodes into which individual ventral roots were drawn. The recorded signals were analog-filtered (DC/0.01–3 kHz) and amplified (gain: 1000), digitized at 5–10 kHz (Digidata 1322A) and stored on a computer. In some experiments, the activity of ventral funiculi (VF) and/or ventrolateral white matter funiculi (VLF) was also recorded. Episodes of data were analyzed off-line using Clampfit. Locomotor activity was quantified using wavelet analysis (Mor and Lev-Tov, 2007). For this purpose, the VR recordings were high-pass filtered at 50 Hz, half-wave rectified, and low-pass filtered at 5 Hz to obtain the envelope of spiking activity. The stimulus artifacts were removed digitally and the rectified integrated neurograms were resampled at 50 Hz.

### Stimulation

To elicit locomotor-like activity, dorsal and/or ventral roots were stimulated with trains (frequency: 1–5 Hz, duration: 5–15 s) of square-wave electrical pulses (duration: 200–250 µs). The stimulus intensity was based on the threshold for eliciting fictive locomotion. The threshold (Thr) for a given spinal root was defined as the lowest current intensity at which that root had to be stimulated to elicit locomotor-like activity in 5/5 attempts. Typically, the threshold for dorsal roots (L6–S3) was 10–15µA and for ventral roots (L4–L6) it was 20–35µA. The range of stimulus intensities used in our experiments was 0.8–10 times the threshold (0.8–10× Thr). Descending pathways from the brainstem were excited in the same way, using a bipolar stimulation electrode placed in the ventromedial medulla (Zaporozhets et al., 2004; Blivis et al., 2007). To elicit monosynaptic responses in motoneurons or VF/VLF interneurons, the appropriate DRs were stimulated with a single electrical pulse.

### Data analysis and statistics

To quantify locomotor-like activity, we used wavelet analysis (Torrence and Compo, 1998; Grinsted et al., 2004; Wang et al., 2004; Mor and Lev-Tov, 2007). Analysis was performed using modified MATLAB (MathWorks) routines originally developed by Torrence and Compo (1998) and Grinsted et al (2004) and adapted for electrophysiological analysis by Mor and Lev-Tov (2007). The code is freely available for download at https://github.com/avinashpujala (The relevant code is in the repositories “General” and “Spectral-Analysis”). Tutorials for usage of scripts will be provided upon request.

The wavelet transform (WT) decomposes a signal into a set of “wavelet” coefficients using a damped and finite function of zero mean, called the mother wavelet. This function is localized in time and frequency. Here, we used the “complex Morlet wavelet”, which is obtained by modulating a sine wave with a Gaussian (Mor and Lev-Tov, 2007).

Cross-wavelet transforms (XWT) were computed to assess the relationship between pairs of time series (Grinsted et al., 2004; Mor and Lev-tov, 2007) as follows:

(1) $$W_{x}{}_{y}=W_{x}W_{y}^{*}$$

Here, $W_{x}$ and $W_{y}$ are the wavelet transforms of the two time series x and y and * denotes the complex conjugate. As with the entries in $W_{x}$ and $W_{y}$, the entries in $W_{xy}$, termed cross-wavelet (XW) coefficients, are also complex numbers. Thus, they can be represented as vectors in the two-dimensional orthogonal space spanned by the real (abscissa) and imaginary (ordinate) number lines. Because the different rows *(i)* and columns *(j)* of $W_{xy}$ correspond to different frequencies (or scales) and times respectively, each coefficient of $W_{xy}$ corresponds to a unique location in frequency-time space. The vector length of a coefficient provides a measure of the combined power (XW power) between the signals x and y at a specific location in frequency-time space, whereas the vector angle of that coefficient indicates the phase difference between the signals at that same location.

To examine only the locomotor-like activity component of an evoked episode we set phase values outside the range of 180°±60° to zero.

(2) $$W_{x}{}_{y}(i,j)=\left\{\begin{array}{l} W_{x}{}_{y}(i,j),180{}^{\circ}-60{}^{\circ}\leq \arg W_{x}{}_{y}(i,j)\leq 180{}^{\circ}+60{}^{\circ}\\ 0,otherwise \end{array}\right\}$$

The power of the XW spectrum was normalized by the *crossvariance* of the two time series as follows (Grinsted et al., 2004):

(3) $$\frac{W_{x}{}_{y}}{\sigma_{x}\sigma_{y}}$$

Here, $\sigma_{x}$ and $\sigma_{y}$ are the standard deviations (SDs) of the ventral root recordings x and y. This normalization expressed the values of XW power in units of variance and allowed the comparison of spectrograms generated from different preparations. To exclude noise-related activity from the spectrogram displays, we deleted those entries in $W_{xy}$ whose XW power was not statistically different from values expected for background white noise. Significance was established at the 5% level (Grinsted et al., 2004; Mor and Lev-Tov, 2007).

**Figure 1.**
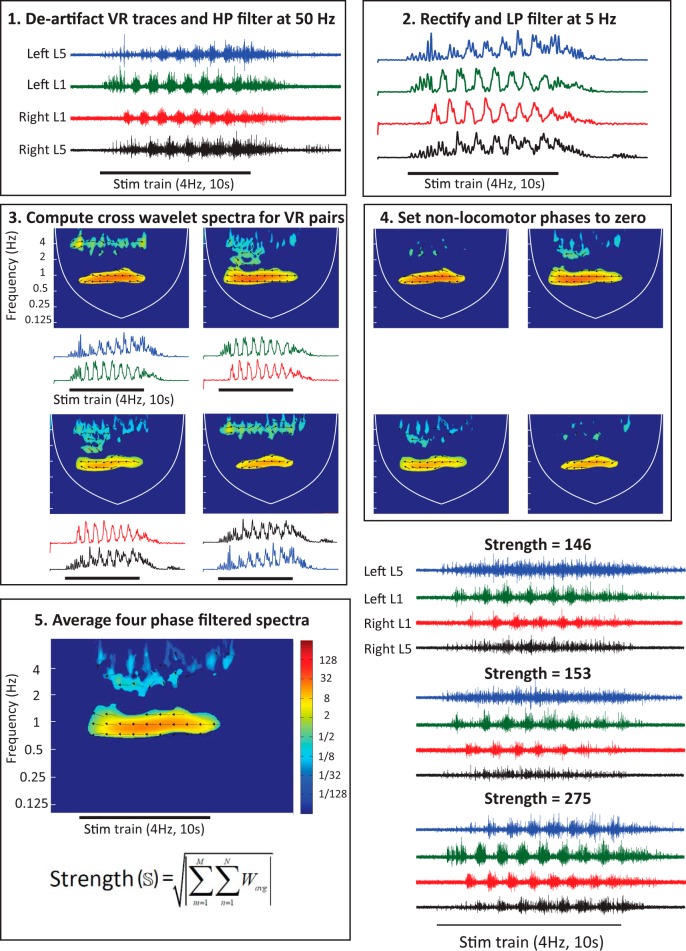
Schematic showing the calculation of locomotor strength. The five numbered panels illustrate successive operations performed on the four VR records. The VR records are first high-pass (HP) filtered at 50 Hz and the stimulus artefacts digitally removed (1). After rectification and low-pass (LP) filtering (2), the cross-wavelet spectra are computed (3) for each pair of VRs whose activity is expected to alternate (L5L vs L1L; L1L vs L1R; L1R vs L5R; L5R vs L5L). The non-locomotor phases (>240° and <120°) are then set to zero (4). The four resulting spectra are then averaged (5) and the strength is calculated as indicated by the formula. The power in the cross-wavelet spectrum is indicated by the color map shown to the right of the spectrum. Bottom right, Three locomotor-like episodes are shown with different locomotor strengths. Note the poor development of bursting in the bilateral L5 roots is reflected in the low strengths (146, 153) of the first two episodes. Once the L5 bursting occurs the strength increases to 275.

We quantified episodes of locomotor-like activity by computing the *strength* (S) of the activity as illustrated in Figure 1. We used bilateral pairs of flexor-dominated (L1 or L2) and of extensor-dominated (L5) VRs for these analyses. If we denote the signals from these VRs by flexor Left (fL), flexor Right (fR), extensor Left (eL), and extensor Right (eR) respectively, and then alternating bursting is expected between the signal pairs {fL,eL},{fLfR},{fR,eR},and {eL,eR}. Crossed-wavelet transforms were computed between each of these pairs. Non-locomotor-like and nonsignificant values were removed from the resulting matrices of normalized XW coefficients, WfLeL,WfLfR,WfReR,WeLeR. The four coefficient matrices were then averaged as follows:

(4) $$W_{avg}=\frac{1}{4}(W_{fLeL}+W_{fLfR}+W_{fReR}+W_{eLeR})$$

To compute locomotor strength (S) for given episode of locomotor-like activity, we took the vector sum of all the entries in $W_{avg}$ and computed the square root of the length of the resultant vector. Thus, if $W_{avg}$ were an M×N matrix, then:

(5) $$S=\sqrt{\left|\sum_{m=1}^{M}\sum_{n=1}^{N}W_{avg}\right|}$$

where *m* and *n* are the row and column indices respectively.

The comparison of locomotor strengths for different stimulus intensities (see Fig. 3) or for interleaved dorsal and ventral root stimulus trains (see Fig. 7) was made was in the following manner. In each preparation, the strengths were computed for several episodes of fictive locomotion elicited under the various conditions. Then, the strengths were normalized by dividing them by the mean of the strengths for one of the three conditions. For instance, if $S_{1},S_{2}$ and $S_{3}$ are the sets of strengths computed for trials in the conditions 1, 2, and 3, respectively. The normalization of values in each of the sets was done as follows:

(6) $$\frac{S_{1}}{〈S_{1}〉},\frac{S_{2}}{〈S_{1}〉},\frac{S_{3}}{〈S_{1}〉}$$

Where, $〈S_{1}〉$ indicates the mean of all values in the set $S_{1}$. This procedure was repeated for every preparation, so that every time the average strength for one of the conditions always equaled one. This allowed the data to be pooled across preparations in a way that preserved the strength relationships among the different conditions. Significance testing was done using nonparametric tests. For comparison between two conditions, we used the Wilcoxon rank sum test, and for more than two conditions we used the Kruskal–Wallis test (*p* < 0.05).

The process of calculating locomotor strength in this manner is similar to computing cross-spectral power densities (CPSD) for pairs of VR signals with expected out-of-phase rhythms during locomotor-like activity, then calculating the area under the curve for each of these CSPDs for a range of physiologically plausible frequencies, and finally averaging these areas to obtain a single number. Thus, much like the area under a CSPD, locomotor strength depends on the amplitude of the rhythmic signals, relative to background noise, as well as their spectral composition. However, because of the phase filtering process described in Equation 2, unlike the area under a CSPD, the locomotor strength calculation is not influenced by in-phase rhythmic components in pairs of ventral root signals. Even when factoring only out-of-phase (120°≤ϕ≤240°, where ϕ is phase difference) rhythmic components, locomotor strength further depends on the variability of the phase values. This is because the complex elements of the coefficient matrix ($W_{xy}$) generated for a pair of signals VR signals, x and y, are vector-summed during the process of computing the locomotor strength. Thus, locomotor strength is expected to be larger for a pair of out-of-phase rhythmic signals that show little variation within the range of phase values (120–240°) we defined as locomotor-like activity.

To compare the strength of coupling between pairs of VR signals (see Fig. 4), we first computed the cross-wavelet transforms for all pairs of ventral roots expected to show alternating rhythmic activity during fictive locomotion. However, instead of averaging the resultant matrices of wavelet coefficients (Eq. 4), we computed from each matrix a scalar value p whose magnitude depended both on the amplitude of the discharge, as well as the phase coupling between the two sets of rhythms; better quality rhythms with tighter locomotor-like phase coupling yielded higher magnitudes. For the VR pair $\left\{eL,fL\right\},\;$the scalar quantifier $P_{eLfL}$ would be computed as follows:

(7) $$P_{eLfL}=\sqrt{\left|\sum_{m=1}^{M}\sum_{n=1}^{N}W_{eLfL}^{alt}\right|}$$

where, as in Equation 4, *m* and *n* correspond to the row and column indices within the coefficient matrix $W_{eLfL}$ and the superscript alt on W indicates the filtering of values within this matrix based on their phase (Eq. 2).

After computing P values for all VR pairs and trials that were elicited using identical stimuli during an experiment we sorted each value into one of four groups based on the ventral root pair from which that value was generated. We then standardized these values to make them comparable across preparations by always treating the group corresponding to the ipsilateral flexor and extensor roots {if,ie} as the reference group and dividing all values in all groups by the mean of values in the reference group. This procedure is described by the equation below where the ipsilateral reference roots are on the left.

(8) PfLfR〈PfLeL〉,PfReR〈PfLeL〉,PeLeR〈PfLeL〉,PfLeL〈PfLeL〉

Here, P refers to set of all values within a group and 〈P〉 indicates the mean of all values within that group. So for instance, $P_{fLfR}=\left\{P_{fLfR}^{1},P_{fLfR}^{2},P_{fLfR}^{n},P_{fLfR}^{N}\right\},$ where the superscripts on P indicate the trial number.

## Results

### VR-evoked locomotor-like activity results from antidromic stimulation of motor axons not orthodromic stimulation of sensory axons in the VR

**Figure 2.**
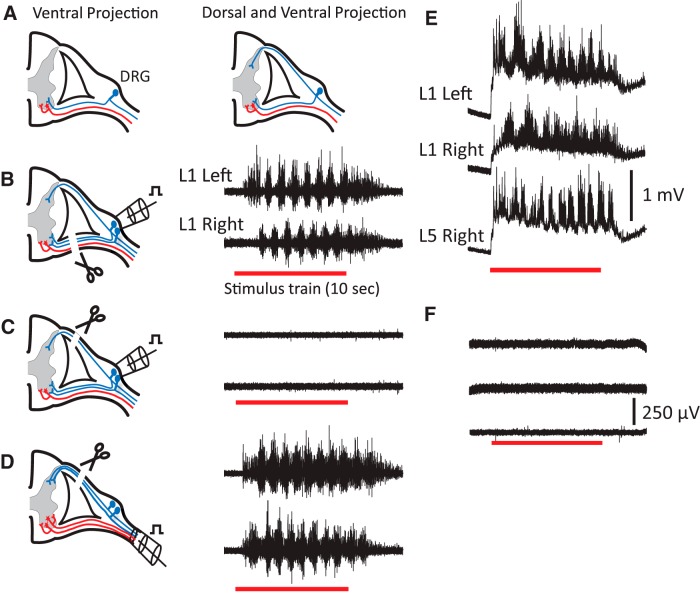
Stimulation of the DRG does not evoke locomotor-like activity if the corresponding DR is cut. ***A***, Schematic to illustrate two possible ways in which DRG afferents (blue) might enter a VR. ***B***, Stimulation of the DRG with the VR cut evokes locomotor-like activity. ***C***, DRG stimulation fails to evoke locomotor-like activity when the DRG is stimulated with the DR cut. ***D***, When the spinal nerve is stimulated with the DR cut, locomotor-like activity is evoked. ***E***, DC recordings of the VRs during an episode of locomotor-like activity triggered by stimulation of the right DRG. ***F***, When the corresponding DR is cut all VR activity is abolished when the DRG is stimulated.

In our earlier experiments (Mentis et al., 2005), we found no evidence of afferent axons in the VRs when the proximal end of a cut VR was back-labelled with fluorescent Dextrans. However, because this method may have failed to identify very small afferent axons in the VRs we used a physiological approach to determine whether stimulation of afferent axons in the VRs was responsible for activating the locomotor CPG by ventral root stimulation. We first established that stimulation of the dorsal root ganglion (DRG) could activate locomotor-like activity with the corresponding segmental VR cut (Fig. 2*B*). The threshold intensity for evoking locomotor-like activity by DRG stimulation was statistically indistinguishable from a dorsal root stimulation suggesting that it was equally effective at recruiting dorsal root afferents (*n* = 20, DRG/DR pairs from 5 preparations; Wilcoxon rank sum test, *p* < 0.05). However, locomotor-like activity could never be evoked when the DRG was stimulated with the dorsal root cut even when stimulating at 10× threshold (Fig. 2*C*).

To maximize the chance of evoking locomotor-like activity in each preparation, we stimulated the DRGs of at least three different segments on both sides of the cord (33 different DRGs from 5 different preparations). We focused on the L4–L6 DRGs because stimulation of the corresponding VRs has been shown to be particularly effective at eliciting locomotor-like activity (Bonnot et al., 2009). Finally, locomotor-like activity was evoked when the spinal nerve was stimulated with a cut dorsal root showing that it was mediated by axons travelling in the ventral root (4 of 5 preparations, 16 of 25 spinal nerves stimulated in total; Fig. 2*D*). In the same experiments, we also used DC recordings of the VR activity to ensure that subthreshold, slow potential activity was not evoked in the VR when stimulating the DRG with the dorsal root cut. As shown in Figure 2*E*, stimulation of the right DRG with its DR intact produced an episode of locomotor-like discharge superimposed on a slow depolarization that briefly outlasted the stimulus train. However, when the DR was cut no slow potential activity was detected in response to DRG stimulation (Fig. 2*F*). Similar results were observed in the other four experiments.

These results, together with the earlier failure to detect VR axons using back-labelling experiments (Mentis et al., 2005), suggest that VR-evoked locomotor-like activity results from the activation of efferents rather than afferents in the VR.

### Properties and comparison of locomotor-like activity generated by dorsal and ventral root stimulation

**Figure 3.**
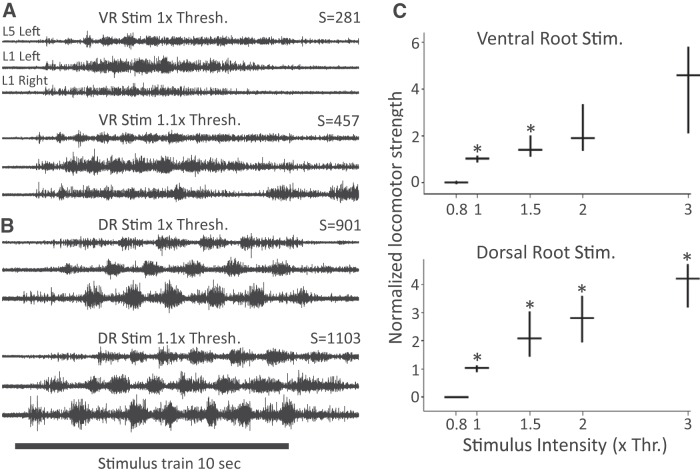
The strength of locomotor-like activity increases with increasing stimulus intensity applied to either a dorsal or a ventral root. ***A***, Locomotor-like activity elicited by stimulation of the L6 ventral root at 1× (top traces) and 1.1× (bottom traces) threshold intensity. ***B***, In the same preparation, locomotor-like activity was elicited by stimulation of the L6 dorsal root at 1× (top traces) and 1.1× (bottom traces) threshold intensity. The bar indicates the duration of stimulus train (10 s). The values of locomotor strength (S; see Materials and Methods) computed for each of the displayed episodes is shown to the top and right of the traces. The displayed values reflect the effect of stimulation intensity. ***C***, Locomotor strengths calculated for dorsal (558 from 63 experiments) and VR-evoked episodes (188 from 24 experiments) are plotted as a function of stimulus intensity expressed in multiples of threshold. The horizontal lines show median values and the ends of the vertical lines mark the 25th and 75th quartile of the sample set. The asterisk above a group indicates a statistically significant difference with the group immediately to the left.

We first examined the relationship between stimulus intensity and the strength of a locomotor-like episode (see Materials and Methods) for both dorsal and ventral root stimulation (Fig. 3). As the stimulus intensity applied to a dorsal or ventral root was increased, a threshold was reached for evoking locomotor-like activity. Below this intensity, locomotor-like activity could not be evoked or was only evoked in a minority of trials. For example, at 0.9× threshold <8% of the trials resulted in locomotor-like activity (2/45 VR stimulation trials from 14 preparations, and 5/70 DR stimulation trials from 23 preparations). When stimulated at intensities up to 3× threshold, the strength of locomotor-like episodes progressively increased (188 VR stimulation trials from 24 preparations, 558 DR stimulation trials from 63 preparations).

We found that even small changes in stimulus intensity could produce substantial increases in the strength of locomotion. In the example shown in Figure 3*A*, when the VR stimulus intensity was increased from 1 to 1.1× threshold, the locomotor strength increased from 281 to 457 and similar effects were observed with DR stimulation.

To quantify and compare the locomotor activity generated by either dorsal or ventral root stimulation, we examined the distribution of the coherent power (normalized to the power of the ipsilateral flexor and extensor discharge) between the following pairs of ventral roots: ipsilateral L1–L2/L5; contralateral L1–L2/L5; bilateral L1–L2; bilateral L5. Using this analysis, a difference emerged between the DR- and VR-evoked episodes (300 trials from 45 preparations for DR stimulation; 145 trails from 25 preparations for VR stimulation).

**Figure 4.**
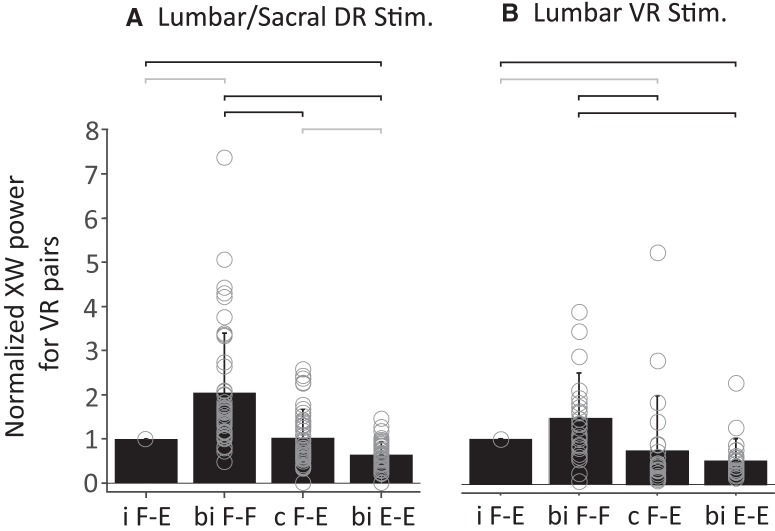
Comparison of the locomotor-like activity produced by dorsal lumbar/sacral root (***A***) and lumbar ventral (***B***) root stimulation. The average coherent power (+SD) between the different root pairs is expressed with reference to the normalized power of the ipsilateral flexor and extensor VRs. For the DR stimulation episodes 300 trials from 45 preparations were used; for VR stimulation 145 trials from 25 preparations were used. The open circles represent the averaged value of the coherent power for each preparation (45 DR, 25 VR). The lines above the graph indicate significant differences between the connected pairs (Kruskal–Wallis followed by χ-squared statistic, *p* < 0.01). The grey lines highlight the significant differences between dorsal and ventral root stimulation. i F-E, L1 versus L5 Ipsilateral to the stimulated root; Bi F-F, left L1 versus right L1; c F-E, L1 versus L5 contralateral to the stimulated root; Bi E-E, left L5 versus right L5.

For both DR- and VR-evoked episodes, the coherent power was greatest between the activity of the bilateral L1 and L2 flexor-dominated roots and weakest between the extensor-dominated bilateral L5 roots (Fig. 4). During DR stimulation, the coherent power between the ipsilateral and contralateral flexor L1/2 and the extensor L5 discharges was similar and weaker than the bilateral flexor coupling. By contrast, when the VRs were stimulated, the ipsilateral flexor/extensor coupling was not significantly different from the bilateral flexor coupling but was significantly stronger than the contralateral flexor/extensor coupling.

### Do motoneuron and DR axons project to the same relay interneurons?

**Figure 5.**
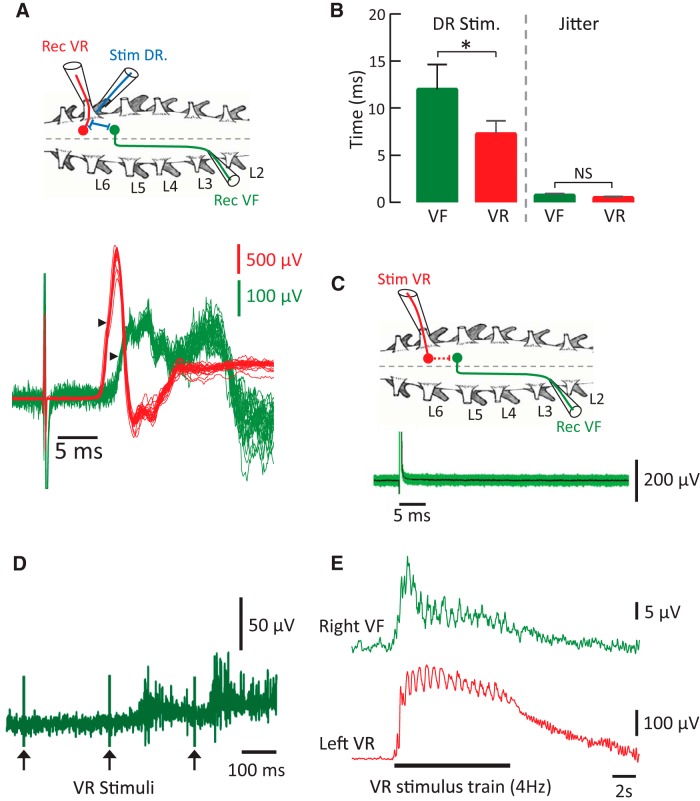
Stimulation of a dorsal, but not a ventral root evokes a short latency potential in the ventral funiculus. ***A***, Schematic of the spinal cord showing the stimulation and recording setup. A stimulating electrode is applied to the left L6 DR (blue) and recording electrodes are applied to the ipsilateral L6 VR (red) and a strip of the contralateral L3 VF (green). Below the schematic are shown 25 superimposed recordings from the VF (green) and the VR (red) in response to DR stimulation (4× Thr) once every 30 s. The arrowheads show the point at which the minimum and maximum latency difference (jitter) was computed. ***B***, The left pair of bars show the mean (±SD) onset latencies of the DR-evoked VR (red) and VF (green) responses, whereas the right pair show the average jitter for each type of recording (6 experiments; 25 trials each). *Indicates statistically significant difference in onset latency (*p* < 0.001, Tukey's multiple-comparisons test). ***C***, Schematic showing the spinal cord with the left L6 VR (red) stimulated and the contralateral L3 VF (green) recorded. The dashed red line within the L6 segment shows the putative monosynaptic connection between the stimulated VR (red) and the cell bodies of the recorded VF interneurons (green). Below this is the response in the VF recording to 100 stimuli (100 µA) applied to the L6 VR every 30 s. The green area defines ±2 SD of the mean. ***D***, Long-latency responses evoked in the VF in response to VR stimuli. ***E***, Simultaneous low-pass filtered VR and VF recordings of a locomotor-episode in response to a VR stimulus train (4 Hz).

It has previously been shown that activation of the locomotor CPG by sacrocaudal afferent stimulation is mediated by relay interneurons whose axons project through the white matter to the lumbar cord (Strauss and Lev-Tov, 2003; Blivis et al., 2007; Etlin et al., 2010, 2013). The projection through the VF is particularly important because cutting this tract compromises activation of the locomotor network by sacrocaudal afferent stimulation (Strauss and Lev-tov, 2003; Blivis et al., 2007; Lev-Tov and O’Donovan, 2009; Etlin et al., 2010; Lev-Tov et al., 2010). The axons of these relay interneurons cross the midline and ascend rostrally, within the ventral funiculus, for several segments. We hypothesized that VR stimulation could activate the locomotor CPG if motor axons also activated these relay interneurons. To test this possibility, we stimulated the dorsal and ventral roots and measured the responses evoked in a strip of the contralateral VF peeled off from the spinal cord ∼3–5 segments rostral to the stimulated roots (Fig. 5).

DR stimulation evoked a short latency potential in both the ipsilateral VR and the contralateral VF. The respective latencies were (7.3 ± 1.4 ms VR, 12.0 ± 2.1 ms VF; 150 trials from 6 experiments). The longer latency of the VF responses to the DR stimulus was because the recording electrode was 3–5 segments from the stimulus location. To establish if these responses were monosynaptic we measured the difference between the minimum and maximum latencies of the responses (defined as jitter) and found that it was statistically indistinguishable for the DR-evoked VR (0.5 ± 0.2 ms) and VF (0.7 ± 0.4 ms) recordings (5 experiments). For comparison, the jitter of monosynaptic EPSPs evoked in muscle identified motoneurons in response to stimulation of the muscle nerve (VRs cut) was 0.4 ± 0.1 ms (Shneider et al., 2009). The slightly longer jitters observed in the present study may be due to introduction of a small delay due to the functional low-pass filtering of that accompanies electrotonically recorded VR and VF potentials. These results suggest that dorsal root afferents project monosynaptically to VF interneurons consistent with earlier immunocytochemical data (Etlin et al., 2013). By contrast, short latency potentials were never recorded from the VF when the VR was stimulated. Indeed, only long latency (74 ± 18 ms, 106 trials from 6 experiments) responses could be detected (Fig. 5*D*) and these could be accompanied by an evoked episode of locomotor-like activity (Fig. 5*E*). In three experiments, we also recorded from the VLF, but were unable to detect any short latency responses following VR stimulation. We conclude, therefore, that motor axons do not make direct monosynaptic connections with the relay neurons that mediate activation of the CPG by DR stimulation.

To establish whether dorsal and ventral root trains activated a common set of postsynaptic neurons we stimulated a dorsal and ventral root pair simultaneously at intensities below their locomotor thresholds to see if the combined stimuli would activate the locomotor network. We reasoned that if the threshold for locomotor activation depended on the recruitment of a critical number of postsynaptic cells, some of which were activated by both dorsal and ventral root stimulation, then stimulating the two roots just subthreshold should recruit enough neurons to trigger locomotor-like activity.

**Figure 6.**
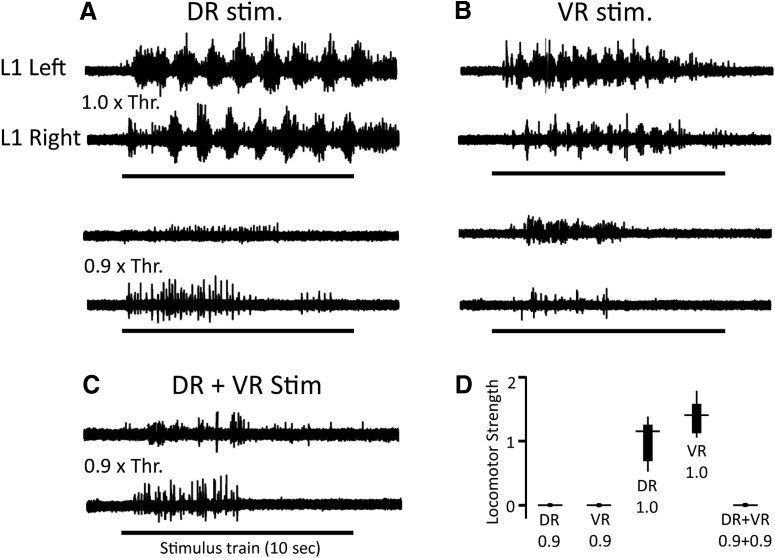
Simultaneous stimulation of dorsal and ventral roots at just subthreshold intensities does not elicit locomotor-like activity. ***A***, ***B***, Top traces show examples of locomotor-like rhythms elicited by stimulation of the homonymous dorsal (left, DR stim) and ventral (right, VR stim) roots at threshold intensity (1× Thr). The line at the bottom of the traces shows the duration of the stimulus train (10 s). The activity was high-pass filtered at 50 Hz. The traces below this show that locomotor-like activity was not produced by root when the stimulus intensity was set to 0.9× threshold. ***C***, When the two subthreshold stimuli were combined locomotor activity was still not evoked. ***D***, Box plots showing pooled data from several experiments (43 trials from 18 preparations). Stimulation of the dorsal and ventral alone or together does not result in locomotor-like activity. The values of strength were normalized to the mean of the ventral root sample set. The lines in the box plots show the median values, and the edges of the black rectangles show the 25th and 75th quartiles of the data.

Before stimulating a pair of dorsal and ventral roots simultaneously, we first established that stimulation of each root individually could activate the locomotor rhythm and that their respective locomotor thresholds remained stable over a 30 min period. If, at any time, the threshold deviated from the initially determined value by >5%, we discarded that root and stimulated a different root. An example of this type of experiment is shown in Figure 6. In this experiment, the L6 dorsal and ventral roots were stimulated initially at the threshold for evoking locomotion (20 µA for VR, 12 µA for DR). Then the stimulus intensity was reduced to 90–95% of this value. We determined experimentally that this stimulus intensity was the highest subthreshold intensity that failed to evoke locomotor-like activity in any of the control trails. When the two trains were delivered simultaneously at this reduced intensity locomotor activity was never evoked. Similar findings were made with other root combinations (Table 1). Indeed, we were surprised that simultaneous subthreshold stimulation of any root pair (DR–VR, DR–DR, or VR–VR) failed to produce locomotor-like activity. These findings indicate that either the two pathways for activating the locomotor CPG did not share early components or that our assumptions concerning the threshold for activation of locomotor-like activity were wrong (see Discussion).

**Table 1.** Pairs of ventral and dorsal roots stimulated to assess spatial facilitation

DR	VR	No.
L5	Ipsi L4	3
	Ipsi L5	5
	Ipsi L6	2
	Contra L5	2
L6	Ipsi L5	3
	Ipsi L6	6
	Contra L6	2
	Contra L5	1
S1	Ipsi L6	3
	Contra L6	2

The various combinations of dorsal and ventral roots are shown with the number of pairs of each class used (No.).

### Afferent, efferent, and descending pathways converge onto higher-order interneurons involved in rhythmogenesis

One limitation of the previous experiments is the possibility that subthreshold stimulation of each pathway fails to propagate beyond the earliest synapses in the pathway. Alternatively, it may be that the initial stimuli in a suprathreshold stimulus train facilitate the pathway allowing the subsequent stimuli to evoke locomotor-like activity. In view of these concerns, we stimulated two roots at suprathreshold intensities but at a frequency (suboptimal) that did not evoke locomotor-like activity. This allowed us to examine the effects of interleaving the suboptimal, suprathreshold stimulus trains from two roots to achieve an effective frequency that was double the individual trains.

**Figure 7.**
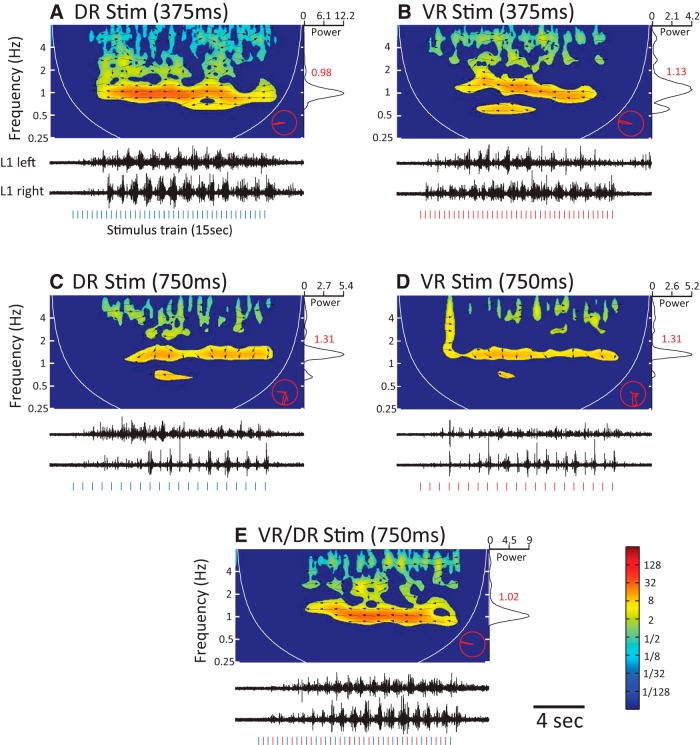
Interleaved, suboptimal dorsal and ventral root-evoked responses combine to trigger locomotor-like activity. ***A***, An episode of locomotor-like activity elicited by stimulation of the left L6 dorsal root with a train of electrical pulses appearing every 375 ms. The black traces show ventral root signals from the left (top) and right (bottom) L1 VRs, whereas the spectrogram above displays the corresponding cross-wavelet power spectrum. The hot regions in the spectrum with leftward pointing arrows indicate locomotor-like activity, while the cooler regions with rightward pointing arrows result from stimulus-locked synchronous responses. The vertical ticks below the VR traces indicate the stimulus pulses. The trace on the right shows the global power spectrum obtained by summing values in the cross-wavelet spectrum along the time dimension for all frequencies. The global power spectrum displayed here has been normalized by the maximum value. The number displayed next to the peak of the global power spectrum (red) indicates the frequency at which the power peaks. The red polar plot in the bottom right corner shows the power-weighted distribution of phase angles (arrows) for all regions of significant power appearing the cross-wavelet spectrogram. ***B***, An episode of locomotor-like activity elicited in the same preparation as in ***A***, but by stimulation of the L6 VR with a train of electrical pulses delivered at intervals of 375 ms (vertical red ticks). ***C***, ***D***, Activity resulting from the stimulation of the aforementioned dorsal and ventral root, respectively, but with pulse trains with an interpulse interval twice as long as in ***A*** and ***B*** (ie, 750 ms). The activity evoked at this stimulus frequency is not locomotor-like, although the bursts in the two VRs appear somewhat rhythmic, their phases are not tightly coupled and are completely out-of-phase. ***E***, When the suboptimal dorsal and ventral root stimulus trains were offset by 375 ms so as to interleave the pulses delivered to the different roots (red and blue vertical ticks below the VR traces) and create a combined train with an effective interpulse interval of 375 ms, locomotor-like activity was evoked.

We first established that stimulation of either a dorsal or a ventral root at 2.67 Hz produced locomotor-like activity that was recorded from the left and right L1 VRs (Fig. 7*A*,*B*). When the roots were stimulated at half this frequency locomotor-like activity was not observed (Fig. 7*C*,*D*), but when these suboptimal DR and VR stimulus trains were interleaved (Fig. 7*E*) locomotor-like activity was triggered.

**Figure 8.**
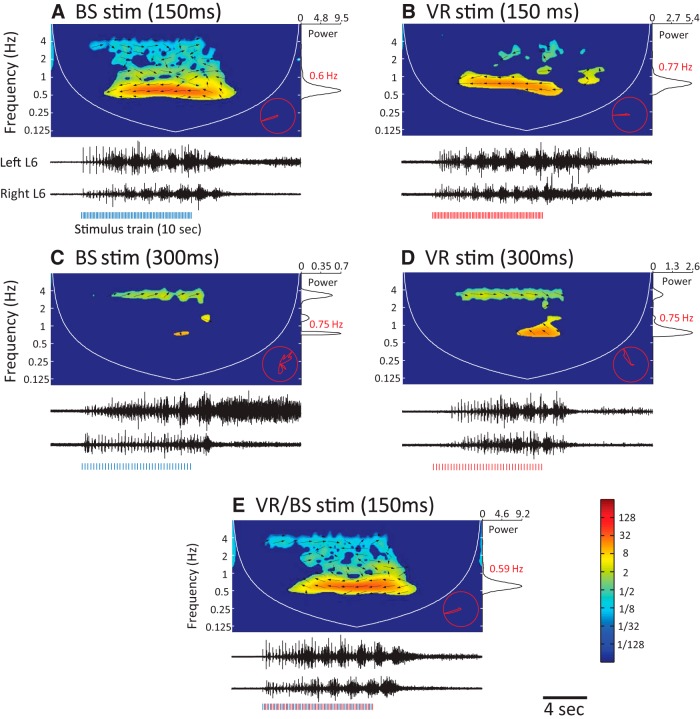
Interleaved, suboptimal brainstem and ventral root stimulus trains combine to trigger locomotor-like activity. The layout of this figure is the same as that of Figure7. ***A***, ***B***, Cross-wavelet spectrograms (top) of locomotor-like activity recorded from the left and right L6 VRs (black traces, bottom) evoked in response to stimulation of the brainstem or the L5 VR with an optimal 6.7 Hz stimulus train. The blue and red bars below the VR activity represent the stimuli. The maximum XW power is at 0.6 Hz (brainstem) and 0.77 Hz (VR) and is shown in the power spectra on the right. The activity between the roots is alternating (arrows to the left, phase plot in the bottom right corner). ***C***, ***D***, Stimulation of the brainstem at the suboptimal frequency of 3.33 Hz evoked synchronized bursting (arrows to the right). Stimulation of the VR at the 3.33 Hz evoked weak alternating bursts together with the stimulus locked synchronous signals. ***E***, Interleaving the 3.33 Hz brainstem and VR stimulus trains (alternating blue and red bars) evoked locomotor-like activity.

We then interleaved the VR trains with stimulus trains applied to the brainstem. Brainstem stimulation is commonly used to evoke locomotor-like activity because it is thought to mimic the physiological activation of the locomotor CPG by descending signals (Atsuta et al., 1990; Zaporozhets et al., 2004; Blivis et al., 2007). As shown in Figure 8, locomotor-like activity was evoked by stimulation of the L5 VR and the brainstem with 6.67 Hz, but not 3.33 Hz stimulus trains (Fig. 8A–D). Although VR stimulation occasionally produced some alternating rhythmic discharge in the bilateral L6 VRs when stimulated at 3.33 Hz (Fig. 8*D*), the overall strength of such activity was much weaker than when the same root was stimulated at 6.66 Hz (Fig. 8*B*). Interleaving the ventral and brainstem stimuli at 3.3 Hz resulted in robust locomotor-like activity.

To quantify the effects of joint, interleaved stimuli we integrated the power in the region of the spectrogram exhibiting locomotor-like activity to obtain a measure of the strength of the locomotor episode. Because all combinations of the stimulated dorsal and ventral pair roots successfully evoked locomotor-like activity, we pooled data after normalizing the samples to account for variation in their median values across preparations (see Materials and Methods).

**Figure 9.**
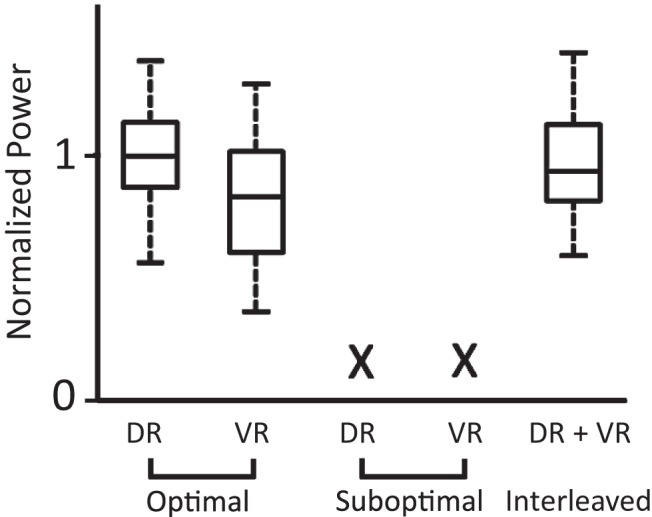
Comparison of the strength of locomotor-like activity evoked by optimal, suboptimal, and interleaved dorsal and ventral root stimulation. Box and whisker plots compare the strengths of locomotor-like episodes evoked when stimulating at the optimal frequency, suboptimal frequency and interleaved suboptimal frequencies. The horizontal lines within the boxes indicate the median values, the lower and upper edges of the boxes mark the 25th and 75th percentile, and the whiskers extend to the most extreme data points. The X indicates that locomotor-like activity could not be elicited. The strength is expressed as normalized values because data from different preparations were pooled. The data were generated from seven ipsilateral pairs of roots and six contralateral pairs.

We found that the strength of locomotor-like activity evoked by dual root stimulation was always within the range of values obtained by stimulation of dorsal and ventral roots alone (Fig. 9), as long as the stimulation frequency was matched in all three conditions (Kruskal–Wallis, *n* = 42, *p* < 0.05). Collectively, these results indicate that the rhythmogenic circuitry common to afferent and efferent pathways is also excited by descending pathways when eliciting locomotor-like activity.

In the final set of experiments, we examined the ability of dorsal or ventral root trains to maintain the locomotor rhythm once it had been initiated by stimulation of the other type of root. If both dorsal and ventral root stimulation activate the same CPG, then once locomotor-like activity was initiated by stimulation of one of the pathways it should continue uninterupted when the stimulus is switched from one pathway to another. To test this prediction, we identified roots that could evoke locomotor-like activity reliably and then stimulated each root at a suprathreshold intensity for eliciting locomotor-like activity with 10 or 15 s stimulus trains (4–5 Hz). From ten such trials we noted the time it took to activate at least one complete cycle of locomotion to insure that the switch in stimulation would only occur once locomotor-like activity has been successfully initiated.

**Figure 10.**
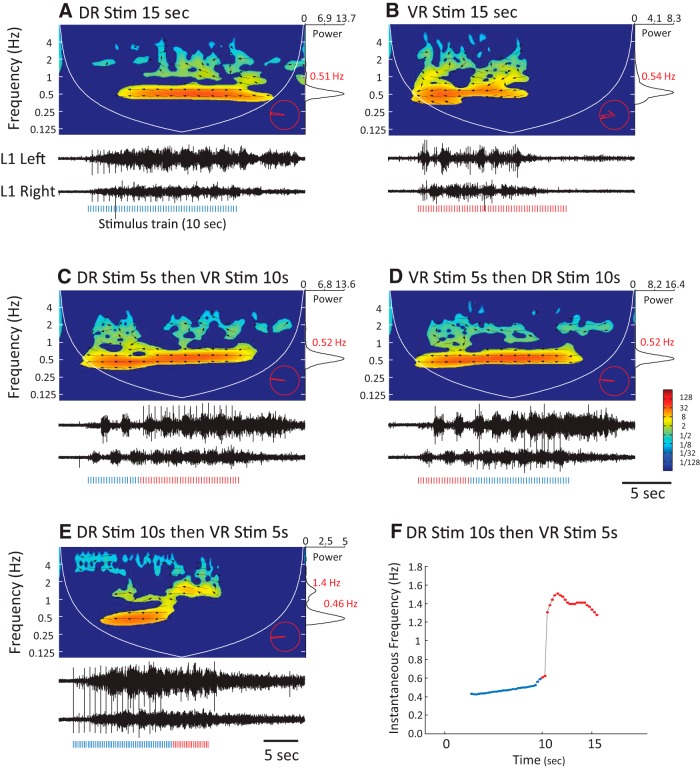
Locomotor-like activity initiated by stimulation of one pathway continues without interruption when the stimulus is abruptly switched to a different pathway. ***A***, ***B***, The panels show episodes of locomotor-like activity recorded from the left and right L1 ventral roots, initiated by either dorsal (A; DR stim 15 sec, 4 Hz, 2 x Thr.) or ventral root (B; VR stim 10 sec, 4 Hz, 2x Thr.) stimulation. ***C***, ***D***, Locomotor episodes evoked by sequential dorsal and ventral root stimulation. ***C***, The DR is stimulated for 5 s (blue stimulus markers below neurograms) followed by VR stimulation for 10 s (red stimulus markers below neurograms). ***D***, The order is reversed with the VR stimulus (5 s) followed by the DR stimulus (10 s). In each case, the locomotor-like episode was evoked at 0.52 Hz. ***E***, Locomotor-like activity evoked by stimulation the right S2 DR for 10 s followed by stimulation of the right L5 VR for 5 s. In this example, the switch in the stimulated root resulted in a rapid change in locomotor frequency from 0.46 to 1.4 Hz without obvious phase perturbation (see phase plot in bottom right corner). ***F***, Instantaneous locomotor frequency for the evoked locomotor episode shown in ***E***.

Figure 10 displays the results of an experiment in which the L6 dorsal root and the ipsilateral L5 ventral root were stimulated. The roots were stimulated separately for 15 s and evoked alternating locomotor-like activity recorded from the left and right L1 VRs (Fig. 10*A*,*B*). We then stimulated each root for 5 s before the stimulation was switched to the other root for the next 10 s (Fig. 10*C*,*D*). We found that rhythmic alternating bursts in the L1 VRs continued to occur without interuption despite the switch in the source of stimulation. As a consequence, the cross-wavelet spectrogram generated from the activity of these VRs showed a continuous region of significant power with locomotor-like phase values (Fig. 10*C*,*D*). In both examples presented here, the switch in stimulation occurred while the right L1 VR was bursting and the left L1 was quiescent but similar results were obtained when the roots were switched in the opposite phase.

In this example, the stimulated dorsal and ventral roots elicited locomotor-like activity of similar frequency. Sometimes, however, stimulation of dorsal and ventral roots elicited locomotor rhythms of different frequencies. In the experiment shown in Figure 10*E*, stimulation of the dorsal root for 10 s elicited locomotor-like activity at 0.46 Hz. When the stimulation was switched to the ventral root for 5 s, the frequency of the locomotor-like activity almost tripled to 1.4 Hz (Fig. 10*F*). This did not depend on the order of root stimulation because the frequency fell when the ventral root was stimulated first. Similar results were observed in 12 experiments (32 dorsal–ventral root pairs stimulated).

## Discussion

We have shown that inputs from the dorsal and ventral roots do not converge at an early point in their respective pathways to the locomotor CPG. Rather, they appear to converge much closer to the CPG and perhaps within the rhythm generating circuitry itself. In addition, the locomotor-like behavior recruited by dorsal or ventral root stimulation is not identical, implying corresponding differences in the composition of the rhythmogenic networks recruited by the different types of stimuli.

Several studies have shown that activation of the locomotor CPG by stimulation of sacrocaudal afferents (Lev-Tov et al., 2000; Whelan et al., 2000) is mediated by a heterogeneous set of relay interneurons that project to the lumbar segments (Strauss and Lev-Tov, 2003; Etlin et al., 2010). Many of these neurons project their axons into the VF where their activity can be recorded in response to sacrocaudal afferent stimulation. Our recordings revealed that lumbar dorsal root stimulation evoked monosynaptic potentials in the VF, consistent with the presence of VGluT1 and VGluT2 afferent terminals on the somata and dendrites of the neurons projecting into the VF (Etlin et al., 2013). However, no such short-latency potentials were recorded when the VRs were stimulated indicating that these cells are not contacted directly by motoneuron terminals.

We were surprised that stimulation of pairs of adjacent spinal roots, just subthreshold for evoking locomotor-like activity, showed no evidence of summation. We assumed that the threshold for evoking locomotor-like activity by either dorsal or ventral root stimulation would depend upon the recruitment of a critical number of postsynaptic cells. Accordingly, combined, just-subthreshold stimulation of a pair of roots would recruit the necessary complement of postsynaptic cells to reach threshold for locomotor-like activity. Because this was never observed for any pair combination (DR–DR, VR–VR, DR–VR), this postsynaptic mechanism for the locomotor threshold appears to be incorrect. Dose et al. (2016) also found that combined subthreshold stimulation of two to three dorsal roots failed to activate the lumbar CPG in the neonatal rat spinal cord which they attributed to the operation of a “gating system that filters the amplitude of afferent stimuli”. However, they provided no evidence in support of this mechanism. It seems unlikely that the locomotor threshold is determined simply by the number of axons activated, because combined stimulation of two roots just subthreshold, while significantly increasing the number of stimulated axons does not trigger the locomotor rhythm. We propose instead that locomotor threshold depends on the species of dorsal or ventral root axons that are recruited by the stimulus. For sacrocaudal afferents it is known that the locomotor CPG can be activated by high threshold mechanical, thermal and nociceptive afferents (Blivis et al., 2007) but not by low-threshold muscle afferents (Etlin et al., 2013). In addition, high-threshold capsaicin-sensitive afferents expressing transient receptor potential vanilloid 1 (TRPV1) have been shown to contribute to the activation of the CPG by sacrocaudal afferent stimulation (Mandadi et al., 2013). Therefore, our inability to observe spatial facilitation between pairs of dorsal roots stimulated at subthreshold intensity may be explained by a failure to activate the appropriate class of high threshold afferent. This does not imply that either the axons or the postsynaptic neurons recruited by the subthreshold stimulus do not contribute to locomotor activity once it is generated. Etlin et al. (2013) split the first coccygeal dorsal root and stimulated one branch at group I–II strength and the other above the threshold for A-δ fibers. Stimulation of the first branch did not evoke locomotor activity whereas stimulation of the second branch did. However, when both were stimulated together the evoked locomotor-like activity exhibited greater power and more cycles compared with stimulation of the second branch alone.

Although the axonal composition of DRs is highly varied, the same is not true for VRs. VRs comprise the axons of somatic motoneurons, γ-motoneurons that innervate muscle spindles and in some segments pre-ganglionic autonomic efferents (Biscoe et al., 1982). Unfortunately, little is known about the recruitability of the various axonal classes as a function of stimulus intensity in the neonatal mouse spinal cord. However, it is unlikely that the recruitment of high threshold preganglionic axons is necessary to trigger the locomotor generator because ventral roots that contain very few preganglionic axons (Biscoe et al., 1982) can evoke locomotor activity when stimulated (Bonnot et al., 2009). These observations raise the possibility that the axons of small α-motoneurons or those from γ-motoneurons have to be stimulated to trigger the activity. γ-motoneurons are known to have recurrent collaterals and also to receive recurrent inhibitory input from both α- and other γ-motoneurons. In contrast to α-motoneurons, much less is known about the synaptic connections of γ-motoneurons within the spinal cord and in particular if their only synaptic targets are Renshaw cells.

An alternative possibility is that stimulation of two roots causes some form of mutual inhibition of the evoked responses. Consistent with this possibility, we found that stimulation of two roots at 0.9–0.95× threshold never evoked a locomotor-like episode whereas stimulation of a single root at the same intensity resulted in locomotor-like activity in 8% of trials. However, we believe this difference to be due to technical rather than biological factors. When subthreshold stimulation of a single root does produce a bout of locomotor-like activity, the observed bout tends to be weak and the locomotor strength tends to have a value close to zero. In other words, bouts of locomotor-like activity elicited by subthreshold stimulation are often barely detectable, whether on the basis of visual inspection of activity in the VR or on the basis of the computed strength. In addition, subthreshold stimulation often evoked disorganized activity and stimulus-locked firing. It is possible, therefore, that simultaneous subthreshold stimulation of a pair of roots increases non-locomotor activity that occludes the detection of what might have otherwise been barely detectable locomotor-like activity. The strength metric is sensitive to such occlusion because non-locomotor-like activity can increase the variance of the signal and thereby affect the significance estimation of simultaneously occurring weak locomotor-like activity. In other words, increased variance can act as noise, and thus decrease the signal to noise ratio. And because significance estimation imposes a threshold such that values below the threshold are set to zero, weak bouts of locomotor-like activity that do not cross the significance threshold may result in strength values of zero.

The basic pattern of locomotor-like activity was similar in episodes evoked by dorsal and ventral root stimulation. Nevertheless, the pattern of activity evoked by each type of stimulus was distinct. At equivalent stimulus intensities (times threshold) VR-evoked episodes generally exhibited a higher frequency of bursting than those evoked by DR stimulation. In addition, the distribution of coherent power between the recorded ventral roots exhibited characteristic features for the two types of evoked episodes. With both types of stimulation, the coupling between the bilateral, flexor-dominated L1/2 roots was much stronger than the coupling between the L5/6 extensor-dominated roots. Clear differences were seen, however, between the coupling of the flexor- and extensor-dominated roots between the two types of stimulation. When the DRs were stimulated, the power was similar for the flexor/extensor coupling on each side of the cord. VR trains, however, produced episodes in which the flexor/extensor coupling ipsilateral to the stimulated root was as strong as that of the bilateral flexor L1/2 coupling and was ∼2× stronger than that of the contralateral flexor/extensor coupling.

The flexor dominance seen in the ventral root recordings might reflect an asymmetry in the operation of the central pattern generator and its outputs. Consistent with this idea, recording of burst deletions show that flexor deletions are accompanied by tonic extensor activity, whereas extensor deletions do not perturb the flexor rhythm recorded from the L1 or L2 segments (McCrea and Rybak, 2007; Zhong et al., 2012). This has led to the suggestion that the rhythm generating component of the CPG is asymmetric with a flexor center that generates the locomotor rhythm and an extensor center that bursts because of rhythmic inhibition derived from the flexor center (Rybak et al., 2015; Shevtsova et al., 2015).

Current models divide the locomotor CPG into a rhythm generating circuit and a pattern generating circuit that controls the timing of motoneuron bursts (McCrea and Rybak, 2007, 2008; Zhong et al., 2012; Rybak et al., 2006a,b,2015; Shevtsova et al., 2015). Accordingly, the differences between dorsal and ventral root episodes might be due to differential activation of components of the pattern generating circuits by the two types of stimuli. However, although this may account for the differences in the strength of coupling between various motoneuron pools it cannot account for the differences in the frequency of DR- and VR-evoked episodes. This difference was particularly evident when the stimulus was switched abruptly from dorsal to ventral resulting in a progressive increase in burst frequency once the VR was stimulated (Fig. 10*E*,*F*). It is known that a subset of the V2a (Zhong et al., 2011; Ampatzis et al., 2014) and V0 (Talpalar et al., 2013) neuronal populations are progressively recruited as locomotor speed increases. Moreover, genetic ablation or acute silencing of V1 interneurons slows the locomotor rhythm (Gosgnach et al., 2006). It is possible, therefore, that the differences in the frequency of DR- and VR-evoked rhythms might be due to the differential recruitment of these interneuronal populations.

Finally, it is important to emphasize that our results cannot be explained by current models of the locomotor circuitry which do not include excitatory inputs from motoneurons. This is largely because the nature of the motoneuron excitatory drive to the locomotor CPG is unknown. One possibility is that motoneurons project to a class of excitatory interneurons in addition to Renshaw cells. This suggestion has been made by Machacek and Hochman (2006) and Humphreys and Whelan (2012) who have provided evidence that the excitatory pathway is enhanced by noradrenaline and inhibited by dopamine. At the present time we do not know the identity of this excitatory pathway or the extent to which it is engaged during drug-induced or normal locomotion.

## Synthesis

The decision was a result of the Reviewing Editor Trevor Drew and the peer reviewers coming together and discussing their recommendations until a consensus was reached. A fact-based synthesis statement explaining their decision and outlining what is needed to prepare a revision is listed below. The following reviewers agreed to reveal their identity: Patrick Whelan, Simon Gosgnach.

The current manuscript extends work performed by the group in 2006 where they were able to demonstrate that activation of the ventral roots could activate locomotor activity. This was an interesting result since it was independent of the Renshaw INs. Here they show that the circuitry does not share common first/second-order interneurons with dorsal root afferents and thus appears to have separate projections onto the CPG. This work provides more data describing this alternative pathway for evoking/modulating locomotor activity.

General Comments

As stated in the last review, this is an interesting study regarding the convergence of dorsal root and ventral root input and the likelihood that they may not share common second-order interneurons. The authors have improved the manuscript since the last submission. There are no further major comments.

Minor Comments.

Is it necessary to mention SpinalCore if no analyzed data is included in the paper? This seems to be a distraction for the reader as it is not clear what exploratory analysis means. I would suggest deleting this.

Pg6 p2,ln 4 Something wrong here, "using and modified"

Pg6, p2,ln6 I suggest changing "software" to "code" or "routine" to distinguish it from the software, "Matlab".

Pg10, p2 The sentence at the bottom of this paragraph beginning, "Thus, locomotor strength ….." needs to be reformulated.

Pg13,p1,ln 7 Fig 2D presumably.

Correct significant digits used in reporting. See page 16 where you move from 2 sig. digits to 1 in your reporting.

Pg27, Please explain the code for the heat map in the legend (part 5).
